# Urban-rural differences in COVID-19 exposures and outcomes in the South: A preliminary analysis of South Carolina

**DOI:** 10.1371/journal.pone.0246548

**Published:** 2021-02-03

**Authors:** Qian Huang, Sarah Jackson, Sahar Derakhshan, Logan Lee, Erika Pham, Amber Jackson, Susan L. Cutter

**Affiliations:** Department of Geography, Hazards and Vulnerability Research Institute, University of South Carolina, Columbia, South Carolina, United States of America; The University of Tennessee, UNITED STATES

## Abstract

As the COVID-19 pandemic moved beyond the initial heavily impacted and urbanized Northeast region of the United States, hotspots of cases in other urban areas ensued across the country in early 2020. In South Carolina, the spatial and temporal patterns were different, initially concentrating in small towns within metro counties, then diffusing to centralized urban areas and rural areas. When mitigation restrictions were relaxed, hotspots reappeared in the major cities. This paper examines the county-scale spatial and temporal patterns of confirmed cases of COVID-19 for South Carolina from March 1^st^—September 5^th^, 2020. We first describe the initial diffusion of the new confirmed cases per week across the state, which remained under 2,000 cases until Memorial Day weekend (epi week 23) then dramatically increased, peaking in mid-July (epi week 29), and slowly declining thereafter. Second, we found significant differences in cases and deaths between urban and rural counties, partially related to the timing of the number of confirmed cases and deaths and the implementation of state and local mitigations. Third, we found that the case rates and mortality rates positively correlated with pre-existing social vulnerability. There was also a negative correlation between mortality rates and county resilience patterns, as expected, suggesting that counties with higher levels of inherent resilience had fewer deaths per 100,000 population.

## 1. Introduction

Coronavirus disease 2019 (COVID-19) appeared in late December 2019 in Wuhan, Hubei Province, China. The severe acute respiratory syndrome coronavirus 2 (SARS-CoV-2) arrived in the United States in January 2020 with the first known COVID-19 case in the state of Washington [1, 2]. The World Health Organization declared COVID-19 a pandemic on March 11, 2020 [3] and on March 13, 2020 the U.S. President declared a national state of emergency [4]. The COVID-19 pandemic initially impacted heavily urbanized regions across the country: New York City, Seattle, Los Angeles, and New Orleans. This was expected given that SARS-CoV-2 is more infectious than the flu virus, and the effective reproduction rate (Rt) of the virus is likely higher in large urban areas due to greater reproductive opportunities for the virus [5, 6]. But, does this pattern hold in a less populated and more rural state such as South Carolina?

This paper examines the diffusion of COVID-19 cases in South Carolina from March 1^st^ to September 5^th^, 2020 and compares the initial temporal and spatial spread (March 1^st^—May 30^th^) to the cumulative impact covering March 1^st^—September 5^th^, 2020. The two time frames specifically compare and contrast the spatial patterns of cases (exposures) and outcomes (fatalities) prior to Memorial Day when mitigation actions were in full force, and from Memorial Day to Labor Day when state mitigation actions were relaxed. First, we investigated the COVID-19 diffusion process and timing related to mitigations in the state. Then we examined how the confirmed cases (case rate/100,000) and deaths (mortality rate/100,000) related to pre-existing county conditions of social vulnerability (Social Vulnerability Index (SoVI®)) and resilience (Baseline Resilience Indicators for Communities (BRIC)) and their variability between urban-rural geographies. Different covariates are tested as to their relationship to the exposures and outcomes.

## 2. Underlying health, vulnerability, and response disparities between rural and urban places

Research confirms health disparities between urban and rural areas not only in terms of risk factors and life expectancy [7] but also in testing and health care capacity [8, 9]. For example, rural residents consistently face challenges of hospital closures and health care provider shortages [10]. The adverse impacts of existing health disparities for people living in rural areas continue to be a major source of concern during preparation for and response to widespread disasters. Rural residents, across all racial/ethnic groups, are at greater risk from the five leading causes of death, including heart disease, cancer, unintentional injury, chronic lower respiratory disease, and stroke, compared with urban Americans [11–13]. Behavioral risk factors for hypertension, diabetes, and COPD such as obesity, poor nutrition, smoking, and alcohol consumption, are also higher in rural areas than non-rural places [14, 15].

Documentation of race-based health disparities is extensive [16]. For example, underserved African American populations face higher HIV incidence, greater maternal and infant mortality rates, and the highest mortality rates across combinations of race and rurality for cardiovascular disease (240 deaths per 100,000), cancer (203.1 deaths per 100,000), and stroke (60.1 deaths per 100,000) [11]. In the Southern states, impoverished rural white communities have been severely hit by the opioid crisis along with high rates of non-communicable diseases driven by conditions such as obesity [17]. These underlying medical conditions (hypertension, obesity, diabetes, cardiovascular diseases) are known underlying factors that increase the risk for severe illness from SARS-CoV-2 [18].

Rural Americans face a unique combination of factors that create disparities in access to health care [19–22]. For example, over 125 rural communities had hospital closures in the past decade and many other rural communities have hospitals that struggle to make ends meet from month to month [7]. Also, rural communities frequently struggle with limited health care workforces and resources under ordinary daily conditions [23]. Rural areas demonstrate a visible and disproportionate lack of medical services, including a paucity of primary care physicians such as family doctors, pediatricians, internists, and dentists [21]. In addition, rural areas have fewer available testing sites in comparison to larger metropolitan cities [24], and when tertiary care hospitals reach their capacity, rural communities experience reduced access to critical care services and other health care resources because of timing and proximity, which further deteriorates the pre-existing health care disparities [23]. The resource constraints and staff shortages in health care affect rural areas’ ability to detect, respond, prevent, and control infectious disease outbreaks [25]. How to distribute the scarce medical resources including personnel, equipment, and services effectively has become an important issue during the COVID-19 pandemic, and rural areas are often not top priorities.

Transportation presents another obstacle to accessing health care. Patients are less likely to travel to see a doctor if they live far from one [21]. Research shows that increased travel time and the perceived difficulty in traveling to see a doctor are prohibitive [26]. Arcury et al. [27] investigated how patients in twelve rural counties in North Carolina traveled to their doctor for regular checkups and follow-up appointments and found that patients in possession of a driver’s license were at least twice as likely to attend appointments than those without one.

The disparities between urban and rural places also exist in the cultural perceptions of health and health care. Rural residents often are reluctant to seek health care or engage in preventive health behaviors than urban populations. For instance, rural residents are less likely to use sunscreen to prevent skin cancer [12]; and undocumented Latino communities working in rural industries such as farming, poultry, and meat production often have no health insurance [17].

Lastly, rural residents are encountering health disparities regarding infectious disease prevention and treatment, especially with limited testing and recovery. According to one study [28], deaths from infectious diseases in urban counties decrease much more rapidly than in rural counties.

### 2.1 Pre-existing social vulnerability and community resilience

The social determinants of health provide useful information on underlying health conditions affecting population vulnerability especially to COVID-19 [24, 29]. Social vulnerability emphasizes a condition rooted in historical, cultural, social, and economic processes that affect individuals’ or society’s ability to cope with and respond to disasters. Cutter et al. [30] suggest that social vulnerability is the product of social inequalities and place inequalities. The use of social vulnerability metrics helps to identify the pre-existing levels of social vulnerability in communities or counties and can assist in highlighting or targeting areas in need of enhanced preparation or response to disasters, including pandemics.

One well-known tool is the Social Vulnerability Index (SoVI), an empirical place-based composite metric that compares social vulnerability among places—counties or census tracts. Social vulnerability is a multi-dimensional construct, where the underlying factors intersect to spatially highlight areas with more socially vulnerable populations. To construct the index, 29 input variables known to influence the ability of populations to prepare for, respond to, and recover from adverse events were culled from the U.S. Census American Community Survey (ACS) 5- year data (2014–2018) for all U.S. counties. Using principal components analysis (PCA), eight factors were generated and then summed using equal weighting to produce the overall score for each county. In the most recent version of SoVI (2014–2018), these eight factors explained 77.9% of the variance in the data (S1 File). Given our focus on county-level patterns, not individual risk factors, using such an aggregate index seems prudent given the multi-dimensional nature of social vulnerability. Mapping the aggregate score using standard deviations from the mean visually shows comparisons between counties, our unit of analysis. SoVI has been extensively used in hazards and disaster research and is now an operational tool for emergency managers in preparedness planning and long-term recovery [31, 32].

Community resilience is another perspective on a community’s pre-existing capacity to cope with natural hazards and disasters and several instruments have been proposed for measuring and tracking resilience [33, 34]. The Baseline Resilience Index for Communities (BRIC) was adopted in this study for comparing the inherent resilience of communities with COVID-19 data [35, 36]. The BRIC scores were measured using a capitals approach to community reliance by incorporating measures of the six major capitals known to influence resilience: human well-being/cultural/social; economic/financial; institutional/governance; infrastructure/built environment/housing; community capacity, and environmental/natural (S2 File). The value for each of the six capitals is an average of normalized indicators in that category, varying from 0 to 1, thus the total BRIC score is a value between 1 to 6 (low to high resilience). According to one national study using BRIC, there are significant differences between urban and rural U.S. counties in terms of overall disaster resilience and the underlying factors contributing to it [37].

## 3. Research design and methods

### 3.1 Data

Data sources in this study include (1) publicly available data from *The New York Times* GitHub for COVID-19 confirmed cases and fatalities [38], (2) U.S. Census Bureau data for county-level demographic and geospatial information [39, 40], (3) county population data extracted from USAFacts [41], (4) 2018 Social Vulnerability Index values for South Carolina counties, and (5) county-level BRIC scores [35, 36].

COVID-19 data reported through *The New York Times* Github provides daily cumulative counts of cases and fatalities for all U.S. counties based on compiled reports from state and local health agencies. Confirmed cases include those proven with a positive laboratory test for SARS-CoV-2 RNA and subsequently reported by a government agency. Confirmed death counts include individuals who have COVID-19 listed as their cause of death [38]. For South Carolina, the cases and deaths are reported by patients’ place of residence [42]. Most state health departments only reported confirmed cases, but on April 14, 2020 the CDC provided criteria for including probable cases. Probable cases are based on a combination of evidence from clinical (symptoms), epidemiologic (close contact with a confirmed or probable case), or serological testing (antigen test in a respiratory sample) but without a confirmatory laboratory RNA test [43]. Our source data includes both confirmed and probable cases as currently available.

In the spatial and temporal analyses, unadjusted daily cases and deaths were aggregated based on epidemiological (epi) weeks for each county in South Carolina from March 1st—September 5^th^, 2020. In correlation and regression analyses, the cumulative case rate and mortality rate over the entire study were used, so each county had a single observation with cases and fatalities normalized as confirmed and/or probable cases (# cases per 100,000) and mortality (# deaths per 100,000).

### 3.2 Spatial scan statistic

Spatial Scan Statistic (SaTScan) version 9.6 was used to identify space-time clusters of confirmed cases and fatalities in South Carolina from the initial period to the start of summer (March 1^st^ to May 30^th^ or epi weeks 10–22), compared to the entire study period of March 1^st^ to September 5^th^ (epi weeks 10–36) [44]. For the discrete scan statistics, the centroids of counties are used for the coordinates of cases and the space-time permutations model is applied to detect the clusters. In the space-time permutation model, the case numbers or fatalities are compared to an expected value if the spatial and temporal locations of all cases were independent of each other. The clusters of relatively higher proportions of cases and lower proportions during the study period are reported.

### 3.3 Policy timeline

Using Google’s News search engine, a search of periodicals related to *South Carolina coronavirus* revealed a number of documents concerning various state and local pandemic- mitigation mandates. The document search was conducted between July 2^nd^ and September 15^th^, 2020. Several documents provided collated timelines listing certain jurisdictions and locales. The primary challenge and limitation of using this methodology lie in the delay between the action, or the mandate, and the dates of data collection. Many news articles also refer to abstract dates relative to the original posted date (e.g., “On Monday” instead of “June 1^st^” or “June 8^th^”), making it difficult to decipher whether the text refers to the prior week or an upcoming one. This problem is compounded especially when the article provides a “last edited” date without specifying which part of the text was edited. Much pertinent information is likely left uncollected or not reported, but we maintain that this component of the methodology is supplementary and contextualizes the findings.

### 3.4 Rural-urban classification

South Carolina has a population of roughly 5.15 million residents dispersed over 46 counties in the state, with an average population density of 154 people/square mile [45]. The population density is highly variable among the counties, ranging from 575 persons/square mile in Greenville County to 26 persons/square mile in Allendale. The state is racially and ethnically diverse, with 27% of its residents of African American descent. The proportion of African Americans varies regionally within the state with the largest proportion found in the more rural coastal plain counties from Allendale (73%) to Williamsburg (64%). Nine counties are majority-minority (>50%) in their racial composition (Allendale, Bamberg, Fairfield, Hampton, Lee, Marion, Marlboro, Orangeburg, and Williamsburg).

**Fig 1:**
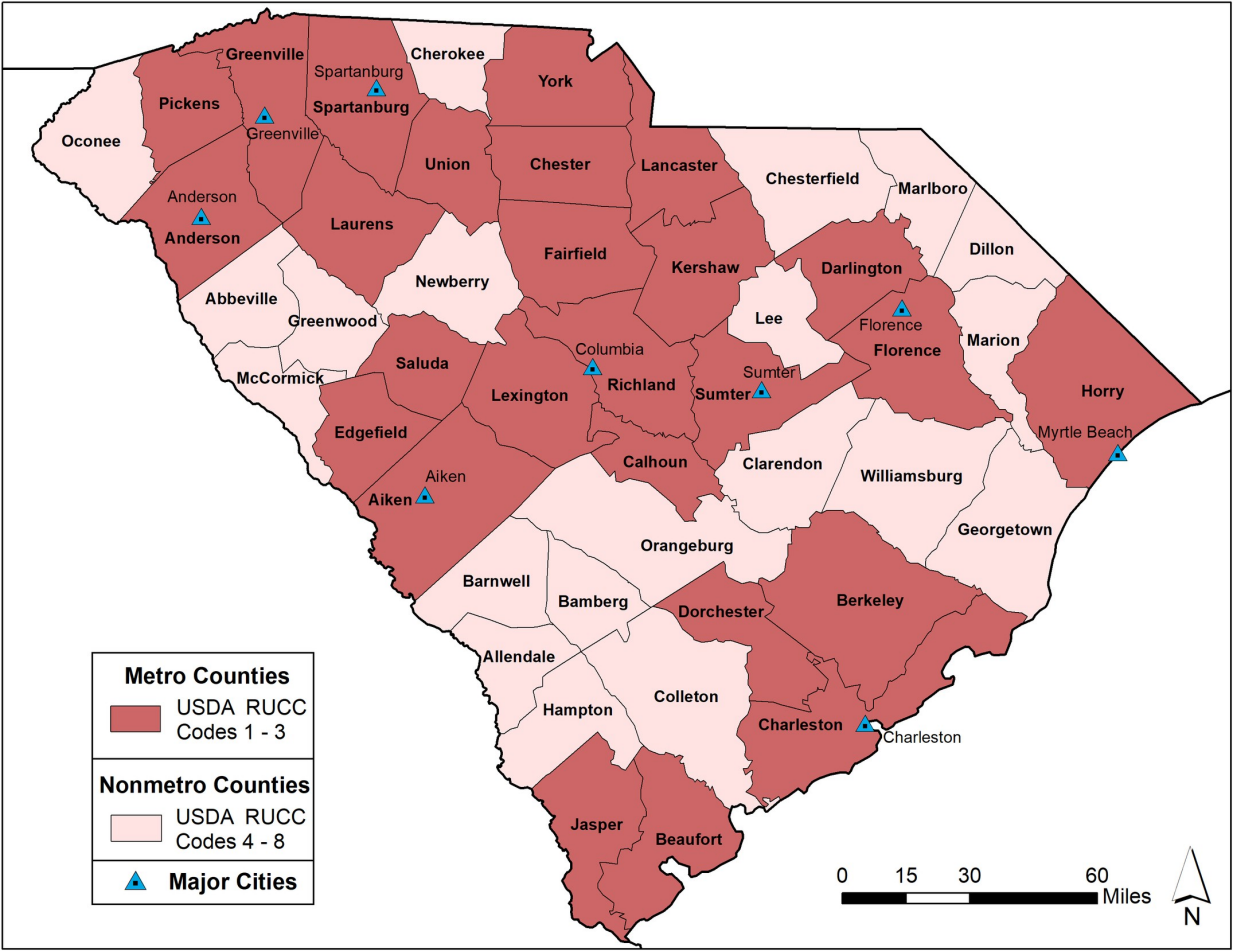
USDA 2013 metro and non-metro South Carolina county designations. County and state boundaries are retrieved from the U.S. Census Bureau (https://www.census.gov/geographies/mapping-files/time-series/geo/carto-boundary-file.html).

To differentiate between rural and urban geographies, South Carolina counties were classified according to the 2013 USDA Rural-Urban Continuum Codes (RUCCs) [46, 47]. The RUCCs designate a code for each county based on a nine-level urban-rural continuum scale, delineated using population thresholds and proximity to a Metropolitan Statistical Area (MSA). An MSA includes the central counties of an urbanized area along with any adjacent counties socio-economically linked to the urban core. The USDA RUCC codes are grouped into a metro (or urban counties) category (codes 1–3) and non-metro (or rural) category (codes 4–9). Among the forty-six counties in South Carolina, twenty-six counties are designated as metro (urban) and the remaining twenty were considered non-metro (rural) (Fig 1).

### 3.5 Statistical analysis

IBM Statistical Package for the Social Sciences (SPSS) Version 25.0 and the ‘spdep’ package in R version 1.3.1093 were used in the analysis [48]. Government mandates were categorized in binary form to represent if a county or cities within it implemented any additional restrictions on people or businesses during the study period (1) or if the county or cities within the county did not implement any restrictions on people or businesses (0). Likewise, the location variable was created using the urban and rural classifications from the USDA codes (1–9) for counties that in turn were classified as either urban (1) or rural (0). Descriptive statistics suggested case and mortality rates in South Carolina exhibited non-normality which was subsequently confirmed using the Shapiro-Wilk test. As a result, the Spearman’s Rank correlation coefficient was used.

A multivariate regression (ordinary least squares [OLS]) initially provided baseline correlations between case rates and death rates, respectively, with SoVI scores, BRIC scores, government mandates, and location (urban/rural classification) used as the correlates. COVID-19 case rates and death rates in each county over the entire study were processed by log10(x) transformation to meet the assumption of normal distribution in OLS. An α = 0.05 was considered as statistically significant in the regression analysis. The model residuals were submitted to spatial dependence analysis by global Moran’s I statistics to assess the need to incorporate a spatial component of the regression model, according to the decision model proposed by Luc Anselin [49]. Once processed, Lagrange multiplier tests were applied to define whether the most appropriate spatial model for the data set would be the spatial lag model or the spatial error model [49]. Finally, residuals from spatial models were subjected Moran’s I statistics to verify that the spatial dependence was controlled. Moreover, the Akaike information criterion (AIC), Wald Statistics, log likelihood, and determination coefficient (R^2^) were used to assess the quality of the final model.

To first explore the differences between urban and rural counties we conducted the Mann-Whitney U two sample t-test against case and mortality rate, SoVI, and BRIC. This non-parametric test allows us to identify significant differences in the mean ranks between the case and mortality rates of COVID-19 in urban and rural counties, as well as the underlying social vulnerability and community resilience in those places. A type-1 error rate of .05 was used to identify statistically significant results in the analysis. However, there were no additional formal statistical procedures (e.g. Bonferroni correction) done to reduce the likelihood of a type-1 error (a false positive result) in the comparisons between urban and rural counties in their case rates or mortality rates.

## 4. Results

### 4.1 Spatial and temporal patterns

**Fig 2:**
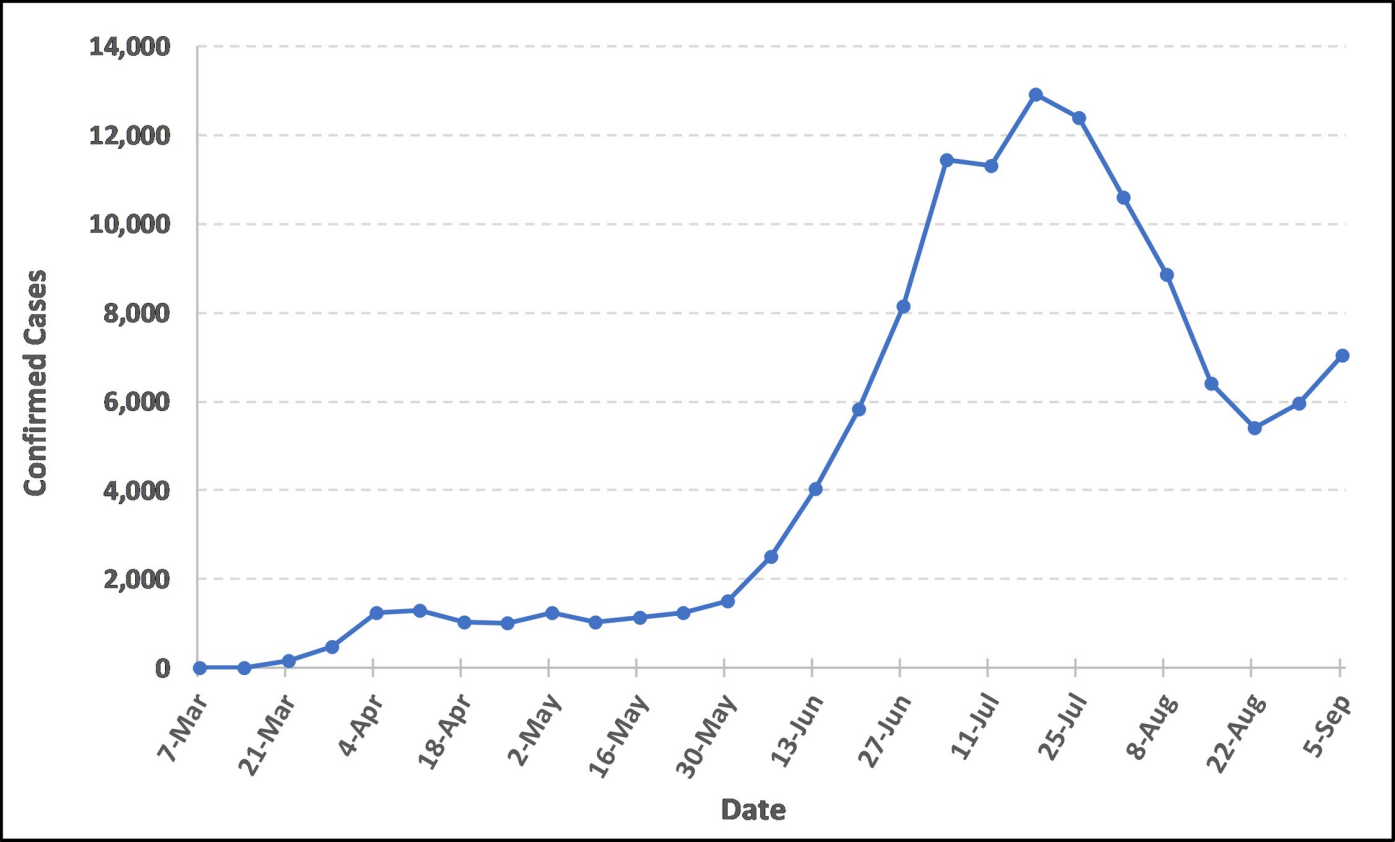
New South Carolina COVID-19 confirmed cases per epi week (March 6^th^–September 5^th^, 2020).

The first objective of the paper was to identify the spatial and temporal patterns of COVID-19 cases in South Carolina and their diffusion during our period of study. The first two cases of COVID-19 in South Carolina were recorded on March 6^th^, 2020 (epi week 10), when two women tested positive—one from Kershaw County and one from Charleston County [50]. As of May 30^th^, 2020 (epi week 22), there were 11,394 total confirmed COVID-19 cases and 487 fatalities across the state. Newly confirmed cases remained under 2,000 until epi week 22, but then exponentially increased during the early summer (Fig 2) and peaking in epi week 29 (July 12^th^—July 18^th^) when 12,913 new cases were identified.

**Fig 3:**
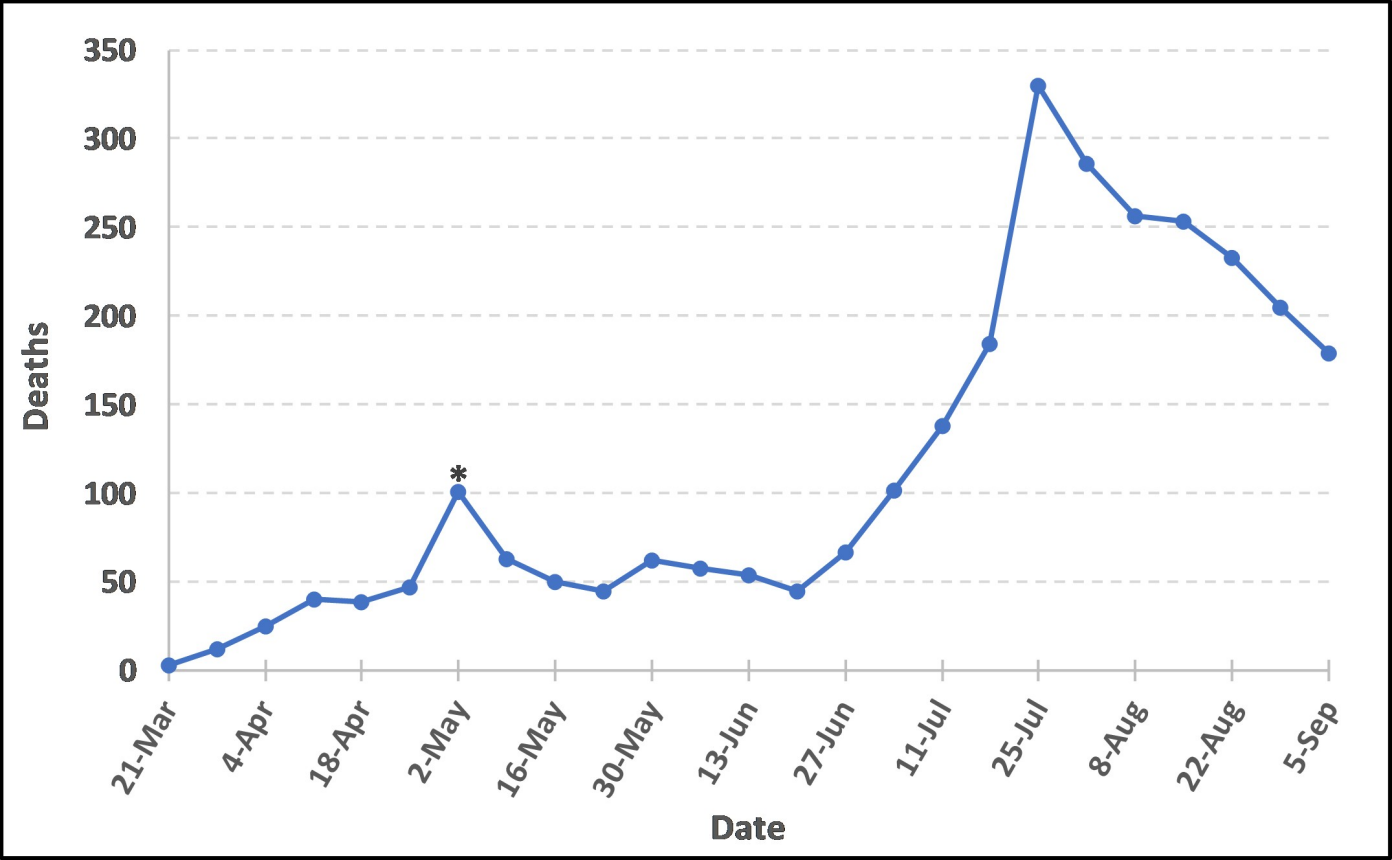
New South Carolina COVID-19 deaths per epi week (March 6^th^–September 5^th^, 2020). The * is a reporting artifact due to technical computer system delays in Electronic Laboratory Reporting to the state. The estimated number for the week is around 72.

The first reported deaths due to COVID-19 in South Carolina occurred during March 15^th^– 21^st^ (epi week 12), with three elderly individuals succumbing to the disease. The number of deaths rose slowly and generally remained under 100 per week until June 28^th^—July 4^th^ (epi week 27). The growth in new deaths continued in the summer, peaking in late July (July 19^th^–July 25^th^ in epi week 30) when 330 deaths were reported (Fig 3). By September 5^th^, 2020, the total confirmed cases increased to 124,289 and cumulative fatalities jumped to 2,877.

**Fig 4:**
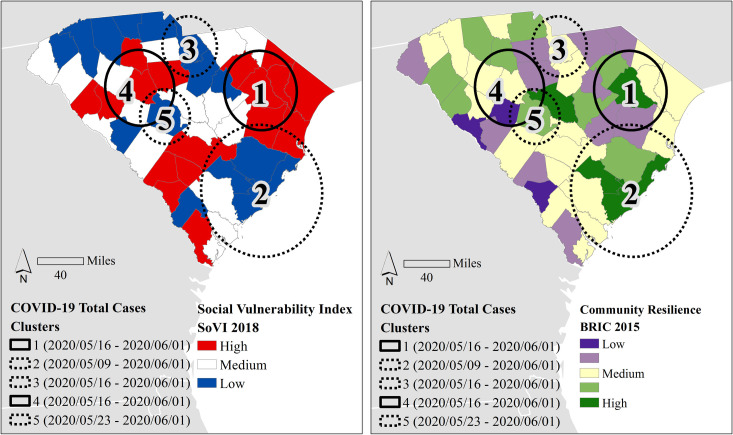
The results for the timeframe of space-time analysis clustering for total cases from the first case in March until Memorial Day, the unofficial beginning of summer (May 30). Using the county’s social vulnerability (left) and community resilience (right) as backdrops, high clusters are denoted by solid lines, while dashed lines refer to low clusters. All clusters are significant (*p* < .001) except for cluster 5. County and state boundaries are retrieved from the U.S. Census Bureau (https://www.census.gov/geographies/mapping-files/time-series/geo/carto-boundary-file.html).

The results of the SaTScan analysis indicate five distinct space-time clusters using the cumulative number of cases. All clusters are statistically significant (*p* < .001) except Cluster 5 (*p* = .011) around Lexington County (Fig 4). The clusters with the highest number of early (May 16–30) cases (Clusters 1 and 4) are in two distinct rural areas within the state. Cluster 1 in eastern South Carolina is in one of the most socially vulnerable regions in the state. The other early cluster of high numbers of cases centered in Greenwood is in an area with medium to medium-high levels of social vulnerability. The earliest cluster of low numbers of cases is around Charleston (Cluster 2), followed by low clusters roughly a week apart—Cluster 3 around Kershaw and Lancaster counties starting May 16^th^ and Cluster 5, Lexington beginning May 23^rd^. All three early low clusters of cases are in counties with low social vulnerability and higher levels of community resilience. The analysis of the total number of deaths in these time frames (March 6^th^ to May 30^th^, 2020) did not identify any statistically significant clusters.

**Fig 5:**
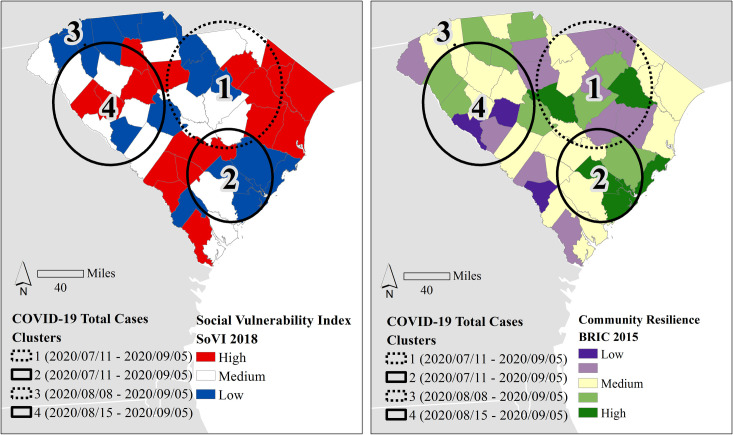
Space-time analysis clustering results for cumulative total cases, March 1^st^ through September 5^th^ (epi weeks 10–36). Using the county’s social vulnerability (left) and community resilience (right) as backdrops, the straight lines refer to high clusters, and dashed lines refer to low clusters. All clusters are significant (*p <* .*001)*. County and state boundaries are retrieved from the U.S. Census Bureau (https://www.census.gov/geographies/mapping-files/time-series/geo/carto-boundary-file.html).

When the time frame expands to Labor Day Weekend (September 5^th^) covering the entire period of study, the results of the space-time analysis in SaTScan indicate a different pattern of case and mortality clustering (Fig 5), including statistically significant clusters (*p* < .001) in deaths compared to the pre-Memorial Day time span. The first cluster of high case counts identified Charleston, Berkeley, Bamberg, Colleton, Orangeburg, and Dorchester counties (Cluster 2) beginning July 11, while the other high case cluster (Cluster 4) started later (August 15) and centered in the Newberry, Greenwood, and Saluda area. One low case count cluster also began on July 11, in the PeeDee area (Cluster 1) near Lee, Sumter, and Darlington counties. The other low case cluster (Cluster 3) centered in Greenville beginning on August 8.

**Fig 6:**
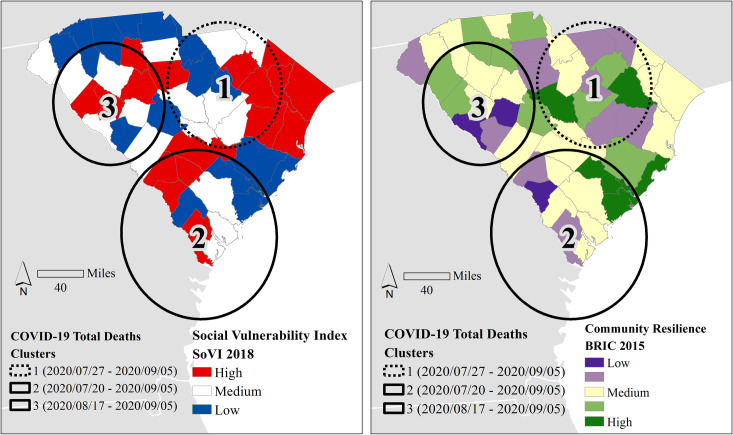
Space-time analysis clustering results for cumulative (a) total cases and (b) total deaths, March 1^st^ through September 5^th^ (epi weeks 10–36). Using county’s social vulnerability (left) and community resilience (right) as backdrops, the solid lines refer to high clusters, and dashed lines refer to low clusters. All clusters are significant (*p <* .*001)*. County and state boundaries are retrieved from the U.S. Census Bureau (https://www.census.gov/geographies/mapping-files/time-series/geo/carto-boundary-file.html).

The SaTScan only identified 3 significant (*p* < .001) clusters of death (Fig 6). The earliest low death cluster (Cluster 1) was identical to case Cluster 1 in Fig 5 in location and extent, only occurring two weeks later. The high death cluster (Cluster 3) also approximated the location and extent of case Cluster 4 in Fig 5, but the timing was nearly identical (beginning on August 17) to the case cluster timing (August 15). The timing of the high death cluster (Cluster 2) was a week later than case Cluster 2 in the same region (Lowcountry), but the death cluster centroid moved south and expanded its areal coverage.

### 4.2 Spread of COVID-19 in relation to state and local response

South Carolina declared a State of Emergency (Executive Order 2020–08) on March 13^th^ (3 days after confirming its first case of COVID-19) [51]. Since this initial declaration, a State of Emergency Executive Order was renewed every two weeks to allow the state access to federal emergency funds. After this initial March 13^th^ declaration, local city and county officials across the state began implementing emergency declarations for their jurisdictions, including actions such as closing schools and non-essential businesses, restricting travel and lodging, suspending certain fees or charges, and enacting Home or Work orders (restricting people to either remain at home or go to work if required, but with limits on other activities). While the state did see an initial steep increase in the number of weekly cases up to April 11^th^ (epi week 15) (Fig 2), as did the rest of the country, these preventative public health actions appear to coincide with an initial decline of cases possibly signaling the beginning of the so-called “flattening” of the curve, at least through the end of April.

However, statewide orders began lifting certain restrictions starting April 20^th^ (epi week 17). These actions included Executive Orders on reopening certain retail stores and public access points. The Home or Work order was lifted on May 4^th^ (epi week 19). Soon after, restaurants (May 11^th^), close-contact operations like fitness centers and public/commercial pools (May 18^th^), and attractions such as zoos, museums, and aquariums (May 22^nd^) were allowed to resume operations following state guidelines, yet no mandatory mask order was implemented at the state level.

While the case numbers slowly increased, approximately two weeks after the start of lifting statewide restrictions and reopening businesses (around Memorial Day weekend), the case numbers began to accelerate. The peak in confirmed cases occurred in mid-July (July 12^th^ -18^th^, epi week 29) when 12,913 cases were recorded and declined thereafter. Four weeks after lifting restrictions, fatalities showed a sharp increase with deaths peaking July 19^th^ - 25^th^ (epi week 30) when 330 deaths occurred (Fig 3).

Despite the lack of a statewide order at the time, local mandatory mask ordinances began in late June to mid-July in many populated cities and towns. Table 1, which is not exhaustive, lists Greenville, Columbia, Spartanburg, Charleston, Hilton Head, Newberry, and North Myrtle Beach, and Orangeburg as some of the cities and towns that implemented additional mask mandates. County mandated mask ordinances began in early July starting with Horry and Georgetown Counties on July 3^rd^ and Richland County on July 6^th^ (Table 1). A statewide mandate requiring masks for entrance to government buildings, restaurants, and restricted gatherings was finally implemented on August 3^rd^.

**Table 1:** South Carolina COVID-19 policies abbreviated timeline.

Timeline Date	South Carolina COVID-19 Policies	Reference
**STATEWIDE**		
**March 13, 2020**	State of Emergency Declaration	[52–54]
	North Myrtle Beach cancels ST. Patrick’s Day parade and festival	
**March 18, 2020**	Curfew effective for Columbia	[55]
**March 28, 2020**	South Carolina receives a federal disaster declaration for COVID-19	[56]
**April 7, 2020**	State "Home or work" order put in place	[57]
**April 20, 2020**	Reopening Executive Order that details reopening certain retail stores while having them to adhere to strict social distancing requirements	[58]
**April 21, 2020**	Reopening Executive Order that removed restrictions on public access points to state beaches, piers, docks, and wharfs with authority given to local officials to restrict access if needed	[58]
**May 4, 2020**	Lift on "home or work" order	[58]
**May 8, 2020**	Boating restrictions lifted	[58]
**May 11, 2020**	Restaurants can choose to reopen for limited dine-in seating at 50% capacity if following state guidelines.	[58]
**May 18, 2020**	Reopening of close contact service providers like fitness centers and public/commercial pools	[58]
**May 22, 2020**	Reopening of attractions like zoos, museums, and aquariums	[58]
**May 28, 2020**	State of Emergency Declaration—Schools to remain closed	[59]
**August 3, 2020**	Statewide mandate requiring a mask or other facial covering in county/municipal courthouses, government buildings, restaurants, and other large venues (e.g. sports stadiums, theatres, concert venues), with additional capacity restrictions.	[60, 61]
**COUNTY/LOCAL**		
**June 23, 2020**	Greenville begins a mandatory mask ordinance	[62]
**June 25, 2020**	Clemson mandatory mask ordinance begins	[62]
**June 26, 2020**	Columbia mandatory mask ordinance begins	[63]
**June 29, 2020**	Spartanburg mandatory mask ordinance begins	[63]
**July 1, 2020**	Charleston, Bluffton, Beaufort, Hilton Head, Newberry, and Mount Pleasant begin following mandatory mask ordinances	[62, 63]
**July 2, 2020**	Lexington and North Myrtle Beach mandatory mask ordinance begins	[62]
**July 3, 2020**	Camden, Sumter, Orangeburg, Horry County, Georgetown city and county begin their mandatory mask ordinance	[62, 63]
**July 6, 2020**	Florence, Conway, and Richland County begin their mandatory mask ordinance	[63]
**July 7, 2020**	Bishopville, Chester, and Irmo begin their mandatory mask ordinance	[62, 63]
**July 10, 2020**	West Columbia, Cayce, and Rock Hill begin their mandatory mask ordinance	[62]
**July 13, 2020**	Manning mandatory mask ordinance begins	[62]
**August 4, 2020**	Columbia extends mask mandate	[64]
**August 10, 2020**	Fort Mill ends mask requirement	[65]
**August 18, 2020**	City of Charleston mask mandate now includes a fine without a warning for not wearing a mask	[66]
**September 4, 2020**	Myrtle Beach extends mask mandate	[67]

Given the known delay between COVID-19 exposure and confirmation of infection with a positive viral test, the decrease in transmission two weeks after the widespread adoption of local masking orders suggests that these orders helped to curb the spread of COVID-19. The weekly confirmed cases started to decrease in epi week 30 (July 19^th^ - 25^th^), and the declining trend continued until epi week 34 (August 16^th^ - 22^nd^), when it began to show a slight uptick in cases as colleges and universities around the state resumed classes.

### 4.3 Urban-rural differences COVID-19 cases and deaths

The second objective of the paper was to ascertain differences between rural and urban counties within the state in terms of cases and mortality. Of the 124,289 confirmed cases between March 1^st^ and September 5^th^, 84 percent occurred in urban counties, unsurprisingly. While the overall statewide cumulative case rate (cases per 100,000) was 2540.21 when adjusting for county-level population size, the COVID-19 case rate showed distinct urban-rural differences with rural county cases rates (2757.40 per 100,000) being higher than those in urban counties (2373.14 per 100,000). When ranking the counties with the highest overall case rates, three rural counties emerged in the top spots (Table 2). Williamsburg County ranked the highest at 4050.21, followed by Lee (4017.11) and Bamberg (3952.79) Counties. Charleston County, designated as urban, held the 4th highest case rate (3498.00) during this time period. Seven out of the ten highest-ranked counties for confirmed cases per 100,000 were rural. However, the Mann-Whitney U test did not confirm statistical significance in the mean ranks of case rates between urban and rural counties (*p* = .080).

**Table 2:** Counties with the highest ranking of confirmed case rates and mortality rates, March 1-September 5, 2020.

Case rates / 100, 000			Mortality rates / 100,000		
County name	Case rates	Urban/Rural	County name	Mortality rates	Urban/Rural
Williamsburg	4050.32	Rural	Bamberg	213.28	Rural
Lee	4017.11	Rural	Lee	190.16	Rural
Bamberg	3952.79	Rural	Clarendon	186.69	Rural
Charleston	3498.00	Urban	Hampton	130.06	Rural
Allendale	3487.57	Rural	Fairfield	129.77	Urban
Orangeburg	3343.20	Rural	Williamsburg	128.42	Rural
Florence	3227.93	Urban	Orangeburg	123.01	Rural
Fairfield	3042.91	Urban	Florence	117.87	Urban
Marlboro	3036.22	Rural	Colleton	103.51	Rural
Clarendon	2963.40	Rural	Calhoun	103.07	Urban

The majority of fatalities from March 1^st^—September 5^th^ occurred in urban counties (81 percent), which was an expected result given the larger population size and greater density. The average mortality rate per 100,000 population in rural counties was much higher (89.94) than in urban counties (58.47), and based on the Mann-Whitney U test, the difference between the mean ranks was statistically significant (*p* = .037). Four of the top five counties with the highest number of deaths per 100,000 are rural (Table 2). Similar to the top ten ranking counties for confirmed cases, counties with the top ten mortality rates also included seven rural counties and three urban counties.

### 4.4 COVID-19 outcomes and their correlates

The third objective of the paper was to describe some of the county-level correlates of COVID-19 case rates and mortality outcomes. Using case rates and mortality rates as outcome variables, we further explored the spatial differences in South Carolina using four explanatory variables—location (urban/rural classification), pre-existing social vulnerability (SoVI), community resilience (BRIC), and presence of local government restrictions (individual county or city ordinances/mandates). After running the descriptive statistical test, additional analyses (correlation analysis, ordinary least squares regression, and spatial lag regression) were conducted to further explore the relationship between case rate, fatality rate, SoVI, BRIC, location, and government restrictions (yes or no).

The mean ranks in SoVI were found to be significantly higher in rural counties compared to urban counties in South Carolina (*p* = .004), using the Mann-Whitney U test. A correlation analysis further confirmed the relationship between SoVI and location was significantly and negatively correlated (Table 3) (indicating a rural association). Social vulnerability had a positive correlation of moderate strength with both case and mortality rates of COVID-19 and the relationship was statistically significant (Table 3). The association between COVID-19 case and mortality rates, rural association, and social vulnerability is important and could indicate that many of the counties heavily impacted by COVID-19 also contain the most vulnerable populations. Six of the ten counties with the highest case rates in the state are identified as having high levels of social vulnerability (Williamsburg, Bamberg, Allendale, Orangeburg, Florence, Fairfield), with Florence and Fairfield designated as urban counties. Similarly, five of ten counties with the highest mortality rates also have high levels of social vulnerability (Bamberg, Fairfield, Williamsburg, Orangeburg, and Florence), again with Florence and Fairfield designated as urban counties.

**Table 3:** Correlates of COVID-19 case rates and mortality rates.

	Case rate	Mortality rate	Location ^a^	SoVI Score	BRIC Score	Government Restrictions
**Case rate**	1	.643**	-.261	.393**	-.227	.036
**Mortality rate**	.643**	1	-.311*	.403**	-.313*	-.242
**Location**	-.261	-.311*	1	-.426**	.502**	.302*
**SoVI Score**	.393*	.403**	-.426**	1	-.278	-.302*
**BRIC Score**	-.227	-.313*	.502**	-.278	1	.351*
**Government Restrictions**	.109	-.251	.341*	-.239	.402**	1

** = p <0.01,

* = p <0 .05

^a^A positive Spearman’s rho with location denotes associations with urban places, while a negative value for location denotes rural places.

Differences in community resilience based on location (urban or rural counties), as well as case and mortality rates were also assessed. BRIC and location were found to have a moderate and significant positive correlation (*r*_*s*_ = .502) at the 99% confidence level. A two sample Mann-Whitney U test between the mean ranks of BRIC in urban and rural counties was also found to be statistically significant (*p* = .001), where urban counties were found to have higher levels of resilience than rural counties. This matches the findings of Cutter et al. [37], which identified this relationship across all counties in the United States. Resilience was also expected to be negatively associated with COVID-19, where less resilient counties would experience higher case rates and mortality rates. Many of the urban counties with the highest levels of community resilience, such as York and Dorchester counties, had lower case and mortality rates per 100,000 people (1,591/19.2 and 2,268/50 respectively). Correlation analysis confirmed this negative relationship between case and mortality rates and BRIC. However, county case rate was not statistically significant while mortality rate was found to be significant at the 95% confidence level. Rural counties with the highest mortality rates, such as Lee and Clarendon, have some of the lowest levels of community resilience in the state. Interestingly, the number of government restrictions in a county was positively and significantly associated with BRIC, where counties with higher levels of underlying resilience issued more mandates on businesses, schools, and travel. However, government restrictions were not associated with case or mortality rates. This may be due to the small number of counties imposing restrictions, their timing (imposing them after rising case rates), and their overall longevity (a few weeks to a few months but inconsistent over the entire study period).

Initially, ordinary least squares (OLS) showed little significance for normalized case rates (R^2^ = .081, *p* = .113) or mortality rates (R^2^ = .053, *p* = .186) based on SoVI, BRIC, government restrictions, or location inputs. However, there was a significant positive relationship between SoVI score and normalized case rates (*p* < .05), but the relationship with other variables was not statistically significant. The relative importance of SoVI was higher in its effect on COVID-19 case rates than BRIC (2.3% to 0.6%), but the opposite was true for mortality rates, where BRIC had more effect (12.0%) than SoVI (3.1%). Also, in case rates, government restrictions (β = -.038) and location (β = -.033) had more effect than SoVI and BRIC combined. Mortality rate is explained by low levels of community resilience (β = -.120) and rural county locations (β = -.080), but the residuals of the OLS models indicated significant spatial autocorrelation (S1 and S2 Tables).

A positive spatial correlation was observed between the normalized case rates and the independent variables (SoVI, BRIC, government restrictions and location) (Moran I = .234; *p* < .05). The normalized mortality rates also had a positive spatial correlation with SoVI, BRIC, government restrictions and location (Moran’s I = .122; *p* < .05). Lagrange multiplier tests and residuals from spatial models indicated the spatial lag model for the normalized cases rates and mortality rates (S2 Table).

The spatial lag model suggested that the normalized case rates were positively associated with SoVI score and government restrictions while negatively associated with BRIC and location (urban/rural classification). The normalized mortality rates were positively associated with SoVI score but negatively associated with BRIC, government restrictions and location. The relative importance of BRIC was the highest in its effect on COVID-19 case rates than SoVI, government restrictions, and location (11.9% to 1.8%, 3.5%, and 1.9%), and same for the mortality rates, where BRIC had more effect (23.4%) than SoVI (2.9%), government restrictions (1.7%), and location (5.1%). None of these parameters are statistically significant (S3 Table). None of the models pass the Breusch-Pagan test for heteroskedasticity, which could be due to a rather small sample size and testing the models for a larger sample might provide different results.

## 5. Discussion

This study is among the first to empirically document significant health inequalities during the COVID-19 pandemic between urban (metropolitan) areas and rural (nonmetropolitan) areas in South Carolina. There are clear temporal and spatial patterns to the progression of COVID-19 in South Carolina culminating in differential impacts between urban and rural counties.

Although the number of confirmed cases and deaths are not associated with local mitigation actions for the entire study period, certain temporal patterns do emerge. While emergency declarations were operative from March through May, the number of cases and deaths were “flat”. However, when certain restrictions were lifted, the rates of confirmed cases and fatalities followed an upward trend beginning after Memorial Day (May 25^th^). Local mandatory mask ordinances in June and July may have assisted in the decline of cases and fatalities in epi weeks 30–34, following the July 4^th^ holiday. The trends could also be due to more testing as the average number of tests also increased from 2,581 per day in epi weeks 10–22 to 6,536 per day in epi weeks 23–26 [38]. There are five early clusters of cases, with two clusters of high cases in largely rural counties, many with higher levels of social vulnerability but with less distinct patterns of community resilience. It is possible that the index cases were randomly transmitted in the rural areas at the early stage when the transmission was unchecked.

Urban areas have a higher total number of COVID-19 confirmed cases, as expected, but lower case rates (per 100,000 population). Condensed residences in urban areas may expedite the SAR-CoV-2 transmission, leading to a higher total number of COVID-19 confirmed cases. However, when standardizing the cases per 100,000 to account for population size, the case rate of COVID-19 from March 1^st^ to September 5^th^ was higher in rural counties (2,757 per 100,000 population) than in urban counties (2,373 per 100,000 population). Despite having a good system of public health clinics strategically placed to serve lower-income and rural communities, the South Carolina Department of Health and Environmental Control (SCDHEC) did not utilize these public health clinics to conduct COVID-19 testing. Instead, they relied on private clinics and hospitals such as CVS and Prisma Health, and free mobile testing events to conduct COVID-19 screening and testing [42, 68]. The heavy reliance on private clinics could make it more difficult to ensure adequate testing sites, testing, and contact tracing to curb virus transmission, especially in rural areas. This is one possible explanation for the higher case rate per 100,000 in rural counties. The impact of case rates (adjusted for population size) may seriously aggravate the existing health care disparities and lead to increased mortality. As shown in our analysis, even though total fatalities in rural areas were lower than in urban areas, the normalized mortality rates in rural areas were indeed higher than in urban areas.

The inherent disaster resilience of counties shows a negative (as expected) but weak and insignificant relationship with COVID-19 case rates, yet a moderate and significant relationship with mortality rate. This indicates that the counties with lower levels of community resilience, which includes the number of hospitals, access to medical care, and hospital beds available, had more COVID-19 deaths. We did find that higher COVID-19 case rates and mortality rates were associated with rural counties and those counties with higher levels of social vulnerability. However, the univariate associations lose their statistical significance when a multivariate approach is taken.

SCDHEC published the South Carolina Infectious Disease plan as Appendix 14–1 to the South Carolina Emergency Operations Plan in January 2017. The Infectious Disease Plan outlined the framework for how they would coordinate the health and medical support during an infectious disease outbreak. Included in that framework was a Pandemic Influenza annex that provided a more detailed description of the roles, responsibilities, and requirements for the various state agencies as well as over 200 specific actions and responsibilities SCDHEC would undertake as the lead agency [69]. Although COVID-19 is not influenza, but rather a novel coronavirus with many different characteristics, the Pandemic Influenza annex provided SCDHEC and other agencies with an existing and detailed response plan to a disaster that no one had predicted. The annex included social distancing measures like quarantining, isolation, and contact tracing of infected individuals, specific reporting requirements for hospital bed occupancy, recommended public school closures, and other actions that we now know are necessary and essential in the response to COVID-19. Although the state developed an infectious disease plan and prepared for a potential pandemic, local governments were not as prepared or misunderstood the threat that a pandemic like COVID-19 posed to their businesses, vulnerable populations, and public health systems. This may partially account for some of the observed urban and rural differences, especially noting the higher levels of community resilience in urban counties.

There were some unique strengths of this study. To our knowledge, this was the first study to examine county-level urban-rural disparities in the COVID-19 pandemic for South Carolina. Additionally, unlike other common descriptive reports, we employed analytical methods to uncover hidden health disparities at county-level scales. This study is not without problems, however. First, there was no socio-demographic information such as race and ethnicity, income, age, and education levels of all COVID-19 patients, deterring our ability to fully examine the disparities at the individual patient scale. However, this problem was not unique to South Carolina as many U.S. states only have aggregated COVID-19 case and mortality data that is publicly available. Nor did we separate out the presence or absence of congregate care facilities that might influence the number of cases and deaths. Second, there were no accurate dates of symptoms, emergency department visits, or hospitalizations for each patient. Therefore, we were only able to use the reported daily confirmed cases and fatalities at the county scale, which we further designated as urban and rural. Third, the COVID-19 surveillance data might be biased due to the limited testing locations in rural areas. Fourth, county units may be too coarse a spatial scale to ascertain linkages between social vulnerability, community resilience, local governmental restrictions, and pandemic outcomes for a relatively small but diverse state. For many urban counties, there are indeed sparse settlement patterns within them outside of the main cities and suburbs. A subsequent sub-county analysis would parse the urban-rural differences more effectively where there would be better spatial alignment with cases and deaths (recorded by Zip Code Tabulation Areas [ZCTA]) and the correlates of social vulnerability and community resilience (recorded at census tract or ZCTA enumeration units). Finally, this study only examined the data for South Carolina, so caution should be exercised in generalizing these findings to other regions especially considering the differences in population structure, socio-economic status, transportation, culture, physical environment, and scale of interventions. If a policy goal is to prevent and prepare for infectious disease, a one-size-fits-all strategy for disaster reduction or mitigation policy actions is inappropriate, since it ignores the inherent variability in capabilities among counties based on the rural-urban character of places. Our findings suggest that the COVID-19 response should have locally customized actions to ensure the greatest success in controlling the disease.

## Supporting information

S1 File Social Vulnerability Index (SoVI). (DOCX)

S2 File BRIC capitals and sample variables. (DOCX)

S1 Table Ordinary least squares- COVID-19 normalized case rates and mortality rates with SoVI score, BRIC score, government restrictions, and urban/rural classification. (DOCX)

S2 Table Ordinary least squares, spatial lag model, and spatial error model results-COVID-19 normalized case rates and mortality rates with SoVI score, BRIC score, government restrictions, and urban/rural classification. (DOCX)

S3 Table Spatial lag model-COVID-19 normalized case rates and mortality rates with SoVI score, BRIC score, government restrictions, and urban/rural classification. (DOCX)

S1 Data (XLSX)
