# Supplementary material for: Urban-rural differences in COVID-19 exposures and outcomes in the South: A preliminary analysis of South Carolina
Source: PLoS One. 2021 Feb 3;16(2):e0246548. doi: 10.1371/journal.pone.0246548 (PMC7857563; doi:10.1371/journal.pone.0246548)
Supplement: S1 File — (DOCX) [file pone.0246548.s001.docx]

**S1 File. Social Vulnerability Index (SoVI).**

The Social Vulnerability Index (SoVI©) is used in this study as a measure of the population's underlying vulnerabilities. Since social vulnerability is conceptualized as a multi-dimensional construct, the social vulnerability index incorporates 29 variables that are calculated for the U.S. counties from 2014-2018 ACS 5-year census data. The standardized variables are placed into a Principal Component analysis (PCA) to generate the multi-dimensional factors that are then evaluated as to their cardinality (+ increases vulnerability; - decreases vulnerability) and then summed to produce the final SoVI score. All factors are equally weighted. See the summary table below and website (https://www.sovius.org).

**Table U.S. County 2014-18 Social Vulnerability Variable and Component Summary**

|  | Percent Asian |
| --- | --- |
|  | Percent Black |
|  | Percent Hispanic |
|  | Percent Native American |
|  | Percent Population under 5 years or 65 and over |
|  | Percent Children Living in 2-parent families |
|  | Median Age |
|  | Percent Households Receiving Social Security Benefits |
|  | Percent Poverty |
|  | Percent Households Earning over $200,000 annually |
|  | Per Capita Income |
|  | Percent Speaking English as a Second Language with Limited English Proficiency |
|  | Percent Female |
|  | Percent Female Headed Households |
|  | Nursing Home Residents Per Capita |
|  | Hospitals Per Capita |
|  | Percent of population without health insurance |
|  | Percent with Less than 12^th^ Grade Education |
|  | Percent Civilian Unemployment |
|  | People per Housing Unit |
|  | Percent Renters |
|  | Median Housing Value |
|  | Median Gross Rent |
|  | Percent Mobile Homes |
|  | Percent Employment in Extractive Industries and Tourism |
|  | Percent Employment in Service Industry |
|  | Percent Female Participation in Labor Force |
|  | Percent of Housing Units with No Car |
|  | Percent Unoccupied Housing Units |

Factors, cardinality and sample variables loading highly on each factor include:

| Factor 1 (+) | **Race and social status** (e.g. Percent Black, Percent female headed household, Percent children not living in 2-parent households, Percent poverty) |
| --- | --- |
| Factor 2 (-) | **Wealth** (e.g. Median house value, Percent earning over $200,000, median gross rent, Per capita income) |
| Factor 3 (+) | **Age (elderly) and dependency** (e.g. Median age, Fewer people per housing unit, Age dependency, Percent with social security benefits) |
| Factor 4 (+) | **Ethnicity** (e.g. Percent Hispanic, Percent speaking English as a second language) |
| Factor 5 (+) | **Special needs populations** (e.g. Hospitals per capita, Percent nursing home residents) |
| Factor 6 (+) | **Tribal communities** (e.g. Percent Native Americans) |
| Factor 7 (+) | **Service sector employment** (e.g. Percent employed in service industry, Percent female participation in the labor force) |
| Factor 8 (+) | **Gender** (e.g. Percent female) |
