# Supplementary material for: Urban-rural differences in COVID-19 exposures and outcomes in the South: A preliminary analysis of South Carolina
Source: PLoS One. 2021 Feb 3;16(2):e0246548. doi: 10.1371/journal.pone.0246548 (PMC7857563; doi:10.1371/journal.pone.0246548)
Supplement: S2 File — (DOCX) [file pone.0246548.s002.docx]

**S2 File. BRIC capitals and sample variables.**

*Human Well-Being/Cultural/Social*—physical attributes of populations, values and belief systems (educational attainment equality, pre-retirement age, personal transportation access, communication capacity, English language competency, non-special needs populations, health insurance, mental health support, food security, access to physicians)

*Economic/Financial*—economic assets and livelihoods (homeownership, employment rate, racial/ethnic income inequality, non-dependence on primary/tourism sector employment, gender income inequality, business size, large retail with regional/national distribution, federal employment)

*Infrastructure/Built Environment/Housing*—buildings and infrastructure (sturdier housing types, temporary housing availability, medical care capacity, evacuation routes, housing stock construction quality, temporary shelter availability, school restoration potential, industrial re-supply potential, high-speed internet infrastructure)

*Institutional/Governance*—access to resources and the power to influence their distribution (mitigation spending, flood insurance coverage, governance performance regimes, jurisdictional fragmentation, disaster aid experience, local disaster training, population stability, nuclear accident planning, crop insurance coverage)

*Community capacity*—social networks and connectivity among individuals and groups (volunteerism, religious affiliation, attachment to place, political engagement, citizen disaster training, civic organizations)

*Environmental/Natural*—natural resource base and environmental conditions (local food supplies, natural flood buffers, energy use, perviousness, water stress)
