# Supplementary material for: Urban-rural differences in COVID-19 exposures and outcomes in the South: A preliminary analysis of South Carolina
Source: PLoS One. 2021 Feb 3;16(2):e0246548. doi: 10.1371/journal.pone.0246548 (PMC7857563; doi:10.1371/journal.pone.0246548)
Supplement: S1 Table — (DOCX) [file pone.0246548.s003.docx]

**S1 Table. Ordinary least squares - COVID-19 normalized case rates and mortality rates with SoVI score, BRIC score, government restrictions, and urban/rural classification.**

| Case Rate | | | | | |
| --- | --- | --- | --- | --- | --- |
| Parameters | | β | S.E. | t | *p* |
|  | Intercept | 3.399 | .548 | 6.207 | .000 |
|  | SoVI Score | .023 | .011 | 2.086 | .043* |
|  | BRIC_Score | -.006 | .208 | -.029 | .977 |
|  | Government Restrictions | .038 | .036 | 1.032 | .308 |
|  | Urban/Rural Classification | -.033 | .041 | -.808 | .424 |
| Mortality Rate | | | | | |
| Parameters | | β | S.E. | t | *p* |
|  | Intercept | 2.157 | 1.126 | 1.916 | .062 |
|  | SoVI Score | .031 | .023 | 1.369 | .178 |
|  | BRIC_Score | -.120 | .428 | -.282 | .800 |
|  | Government Restrictions | -.008 | .075 | -.110 | .913 |
|  | Urban/Rural Classification | -.080 | .084 | -.947 | .349 |

*statistically significant at 95% level of significance

S.E. =Standard Error; β=Beta coefficient estimates
