# Supplementary material for: Urban-rural differences in COVID-19 exposures and outcomes in the South: A preliminary analysis of South Carolina
Source: PLoS One. 2021 Feb 3;16(2):e0246548. doi: 10.1371/journal.pone.0246548 (PMC7857563; doi:10.1371/journal.pone.0246548)
Supplement: S2 Table — (DOCX) [file pone.0246548.s004.docx]

| Case Rate | | | | | | | | |
| --- | --- | --- | --- | --- | --- | --- | --- | --- |
| Model | | Residuals Moran’s I | | R^2^ | AIC | Wald statistics | | Log likelihood |
|  |  | Value | *p* |  |  | Value | *p* |  |
| OLS | Ordinary Least Squares | 3.384 | 0.000 | 0.081 | -63.545 | - | - | - |
|  | Spatial Lag Model | -0.210 | 0.583 | - | -69.288 | 9.387 | 0.002 | 41.644 |
|  | Spatial Error Model | -0.082 | 0.533 | - | -68.621 | 10.725 | 0.001 | 41.311 |
| Mortality Rate | | | | | | | | |
| Model | | Residuals Moran’s I | | R^2^ | AIC | Wald statistics | | Log likelihood |
|  |  | Value | *p* |  |  | Value | *p* |  |
|  | Ordinary Least Squares | 1.930 | 0.027 | 0.053 | 2.763 | - | - | - |
|  | Spatial Lag Model | 0.382 | 0.351 | - | 2.377 | 2.872 | 0.090 | 5.812 |
|  | Spatial Error Model | 0.388 | 0.349 | - | 2.476 | 3.197 | 0.074 | 5.762 |

**S2 Table. Ordinary least squares, spatial lag model, and spatial error model results - COVID-19 normalized case rates and mortality rates with SoVI score, BRIC score, government restrictions, and urban/rural classification.**
