# Supplementary material for: Urban-rural differences in COVID-19 exposures and outcomes in the South: A preliminary analysis of South Carolina
Source: PLoS One. 2021 Feb 3;16(2):e0246548. doi: 10.1371/journal.pone.0246548 (PMC7857563; doi:10.1371/journal.pone.0246548)
Supplement: S3 Table — (DOCX) [file pone.0246548.s005.docx]

| Case Rate | | | | | |
| --- | --- | --- | --- | --- | --- |
| Parameters | | β | S.E. | z | *p* |
|  | Intercept | 1.933 | 0.736 | 2.627 | 0.009* |
|  | SoVI Score | 0.018 | 0.009 | 1.899 | 0.058 |
|  | BRIC_Score | -0.119 | 0.176 | -0.677 | 0.498 |
|  | Government Restrictions | 0.035 | 0.031 | 1.152 | 0.249 |
|  | Urban/Rural Classification | -0.019 | 0.035 | -0.549 | 0.583 |
| Mortality Rate | | | | | |
| Parameters | | β | S.E. | z | *p* |
|  | Intercept | 1.813 | 1.086 | 1.670 | 0.095 |
|  | SoVI Score | 0.029 | 0.021 | 1.399 | 0.162 |
|  | BRIC_Score | -0.234 | 0.390 | -0.599 | 0.549 |
|  | Government Restrictions | -0.017 | 0.068 | -0.254 | 0.800 |
|  | Urban/Rural Classification | -0.051 | 0.077 | -0.663 | 0.508 |

**S3 Table. Spatial lag model - COVID-19 normalized case rates and mortality rates with SoVI score, BRIC score, government restrictions, and urban/rural classification.**

*statistically significant at 95% level of significance

S.E. =Standard Error; β=Beta coefficient estimates
